# Atrial Fibrillation Burden: Impact on Stroke Risk and Beyond

**DOI:** 10.3390/medicina60040536

**Published:** 2024-03-26

**Authors:** Ahmed AlTurki, Vidal Essebag

**Affiliations:** 1 Division of Cardiology, McGill University Health Center, Montreal, QC H3G1A4, Canada; 2 Department of Medicine, Faculty of Medicine, Kuwait University, Jabriya 13110, Kuwait; 3 Division of Cardiology, Hopital Sacre-Coeur de Montreal, Montreal, QC H4J 1C5, Canada

**Keywords:** atrial fibrillation, anticoagulation, burden, subclinical atrial fibrillation

## Abstract

Atrial fibrillation (AF) is an important independent risk factor for stroke. Current guidelines handle AF as a binary entity with risk driven by the presence of clinical risk factors, which guides the decision to treat with an oral anticoagulant. Recent studies in the literature suggest a dose–response relationship between AF burden and stroke risk, in both clinical AF and subclinical atrial fibrillation (SCAF), which differs from current guidance to disregard burden and utilize clinical risk scores alone. Within clinical classification and at the same risk levels in various scores, the risk of stroke increases with AF burden. This opens the possibility of incorporating burden into risk profiles, which has already shown promise. Long-term rhythm monitoring is needed to elucidate SCAF in patients with stroke. Recent data from randomized trials are controversial regarding whether there is an independent risk from AF episodes with a duration of less than 24 h, including the duration of SCAF greater than six minutes but less than 24 h.

## 1. Introduction

Atrial fibrillation (AF) is the commonest sustained arrhythmia encountered in clinical practice [1] and is associated with stroke, myocardial infarction, heart failure and mortality [2,3,4,5,6]. Even in specific settings, such as post operative AF, there is a significant risk of adverse cardiovascular outcomes [7]. Our understanding of AF has progressed over the years, and current evidence clearly demonstrates that a binary definition of AF is not adequate [8]. Interventions for AF have focused on anticoagulation to reduce stroke risk, rhythm, or rate control for quality of life or to improve prognosis as well as treat underlying causes and associated comorbidities [1,9,10,11,12]. In particular, advances in catheter ablation for AF, as well as expanding indications [13], provide an opportunity to address atrial fibrillation burden [14].

Understanding the implications of AF burden is imperative to better target therapies. In this review, we will summarize the current literature evaluating the relationship between AF burden and stroke and cardiovascular outcomes. In addition, we will review the current literature on subclinical atrial fibrillation (SCAF) in the context of AF burden. The effect and role of catheter ablation in ameliorating AF burden are beyond the scope of this review.

## 2. Defining AF Burden

Until recently, AF pattern and, by extension, burden was solely defined clinically based on the form of presentation: paroxysmal if lasting less than seven days, persistent if longer than seven days or requiring cardioversion, long-standing persistent when lasting longer than one year and permanent when rhythm control has been abandoned [15,16]. Some guidelines utilize durations regardless of cardioversion [17]. By moving away from a binary description of AF, the burden of AF provides a more quantitative measurement and can be described simply as the amount of AF [18]. Greater monitoring of AF recurrence provides a much more accurate picture of AF burden [19]. However, even assessing the quantity of AF can take several forms such as the number of episodes or duration of the longest episode [20]; long episodes have been shown to have clinical relevance [21]. Charitos and colleagues have shown that patients within the same clinical classification were highly heterogeneous with regard to AF temporal persistence. In addition, clinical AF classification and objective device-derived assessments of AF temporal persistence correlated poorly [22]. A clinical classification of AF is therefore relatively arbitrary and is hampered by several limitations.

Most importantly, in the absence of long-term rhythm monitoring, classification depends heavily on patient symptoms or random checks with electrocardiogram recordings, which physicians then use to derive a rhythm assumption; this has somewhat improved with wearable devices and portable small rhythm monitors, but these are not yet widely in use. Furthermore, the type and timing of treatment has a significant impact on classification, such as the use of cardioversion or employment of a rhythm strategy in general with the use of anti-arrhythmic drugs [23]. Greater symptoms also predict a greater use of interventions such as cardioversions and ablations [24]. Moreover, significant heterogeneity exists within these groups; both a patient with an hour of AF per year and another with many hours of AF not meeting persistent AF definitions are classified as paroxysmal AF. Similarly a patient with a single ten-day AF episode in a year is classified equivalently to another with a very high daily burden of AF over the course of a year [25]. The most intuitive definition of AF would be the proportion of overall time spent in AF, but this is limited by the need for continuous monitoring such as that employed by cardiac electronic implantable devices [8]. The latter has provided important evidence for our understanding of AF burden [26]. Finding the ideal duration of rhythm monitoring to determine AF burden has been a significant challenge, as most patients do not have continuous monitoring. Aguilar and colleagues, using data from the CIRCA-DOSE [Cryoballoon versus Irrigated Radiofrequency Catheter Ablation: Double Short versus Standard Exposure Duration] study found that shorter monitoring for 24/48 h using electrocardiogram monitors was less sensitive for detecting AF recurrence post ablation and led to overestimation of AF burden when AF occurred. The authors found that the optimal duration of non-invasive monitoring to accurately determine AF burden was 28 days; this could be achieved by applying 7-day monitors four times in a year or by applying 14-day monitors twice per year [27].

Long-term monitoring for AF is important, as clinical AF represents the tip of the iceberg in terms of disease prevalence [28]. This is particularly important in patients with cryptogenic stroke in whom a substantial proportion are found to have underlying AF [29]. Wearable devices and small implantable monitors provide an opportunity to screen for AF. These devices also provide the possibility to assess AF burden. Of the currently available wearable devices, adhesive patches have been utilized the most for evaluating AF burden, particularly after having undergone an ablation procedure [28]. Smartwatch technology using a detection algorithm is another option that can potentially be used to quantify AF burden and has been shown to be able to differentiate AF from sinus rhythm with relative accuracy [93% sensitivity and 84% specificity compared to an electrocardiogram] [30].

## 3. Stroke Risk and AF Burden

Intuitively, one would expect a direct linear relationship between AF burden and stroke risk. However, current guidelines recommend oral anticoagulation based on certain clinical risk factors that have now been established in the literature [12]. This is due to the complex relationship between AF and thromboembolic risk that involves the full spectrum of Virchow’s triad and extends well beyond AF burden [31]. Interestingly, adding AF burden to the typically used score, CHA_2_DS_2_-VASc, was shown to further risk-stratify patients with AF regarding stroke risk and may be potentially useful in guiding anticoagulation therapy [32]. Of note, studies used to derive and validate risk scores for anticoagulation in AF were performed in a patient population with clinical AF [33]. The detection of clinical AF is likely to occur in symptomatic patients or in those with a relatively high burden of AF to be detected by chance [25].

Data on the equivalence of paroxysmal and persistent AF with regard to stroke risk come from early pivotal studies such as the SPAF trial (Stroke Prevention in AF Trial), the ACTIVE-W trial, and the GISSI AF trial. In the SPAF trial, patients with intermittent AF (460) were compared to patients with sustained AF (1552) with no difference in stroke risk at an annual rate of 3.2% and 3.3%, respectively, although the study was non-randomized [34]. The most important limitation is the likely selection of patients with a higher burden of AF in the intermittent AF group to the degree that many may have been classified as persistent using current standards as well as selecting for patients with a higher stroke risk. Data from the ACTIVE-W trial that compared 6706 patients, with paroxysmal versus non-paroxysmal AF, also showed a similar annual risk of stroke at 2% and 2.2%, respectively. However, there were significant rates of oral anticoagulation use in those with non-paroxysmal AF [33]. Analyses from the GISSI-AF trial also showed no difference in stroke risk between paroxysmal and persistent AF [35].

The findings from secondary analyses of contemporary trials of anticoagulation differed from those previously mentioned; these trials were designed for non-vitamin K oral anticoagulants and were non-inferior to warfarin for anticoagulation in patients with AF. In the Rivaroxaban Once Daily Oral Direct Factor Xa Inhibition Compared with Vitamin K Antagonism for Prevention of Stroke and Embolism Trial in Atrial Fibrillation [ROCKET-AF] randomized trial, patients with AF received either rivaroxaban or warfarin. There was a significantly higher risk of stroke (*p* = 0.048) and death (*p* = 0.006) in patients with persistent AF compared to paroxysmal AF [36]. In the Apixaban for Reduction in Stroke and Other Thromboembolic Events in Atrial Fibrillation [ARISTOTLE] randomized trial, in which patients with AF received either apixaban or warfarin, the incidence of stroke or systemic embolism was significantly higher in patients with persistent or permanent AF compared to patients with paroxysmal AF (*p* = 0.015) [37]. Furthermore, an analysis of The Effective Anticoagulation with Factor Xa Next Generation in Atrial Fibrillation–Thrombolysis in Myocardial Infarction 48 (ENGAGE-AF TIMI 48), showed that persistent AF (*p* = 0.015) and permanent AF (*p* = 0.004) conferred an increased risk of stroke compared to paroxysmal AF in the trial comparing edoxaban to warfarin [38]. In a systematic review of 12 studies with almost 100,000 patients, Ganesan and colleagues explored the risk of stroke in patients with paroxysmal versus non-paroxysmal AF. The risk of stroke was significantly higher with non-paroxysmal AF compared to paroxysmal AF (*p* < 0.001); similar results were found when adjusted for confounders (*p* < 0.001) [39].

Importantly, AF burden may aid in stratifying risk within a clinical classification. The KP-RHYTHM study enrolled patients with paroxysmal AF and used wearable monitors for 14 days to define AF burden. After adjusting for CHA2DS2-VASc stroke risk scores [as well as ATRIA scores], the highest tertile of AF burden, which had a burden greater than 11.4%, was associated with a more than 3-fold higher adjusted rate of thromboembolism while not taking anticoagulants compared with lower AF burden groups, and similar findings were found across clinical subgroups [40].

Ablation and AF burden in the context of stroke risk is another important aspect worth considering, with the lingering question of whether anticoagulation can be stopped in patients with low to intermediate clinical stroke risk who have successfully undergone an AF procedure. Proietti and colleagues conducted a systematic review and meta-analysis of 16 studies that enrolled 13,166 patients in whom anticoagulation was stopped and 12,011 in whom anticoagulation was continued post ablation. There was no significant difference in the incidence of stroke and systemic embolism between the two groups, even when stratifying by CHADS_2_ and CHA_2_DS_2_-VASc score and with significantly less bleeding in patients off anticoagulation medication [41]. The Optimal Anti-Coagulation for Enhanced-Risk Patients Post Catheter Ablation for Atrial Fibrillation (OCEAN) trial is an ongoing study that aims to assess if oral anticoagulation is superior to anti-platelets in reducing the risk of stroke and systemic embolism in patients with no documented recurrence of AF post ablation for at least a year [42]. This trial may help identify the optimal long-term anticoagulation strategy after AF ablation and will be reporting results soon [43].

Recent new findings may also contribute to the current body of literature regarding the risk of stroke in AF patients. The use of machine learning and ensemble methods incorporating AF burden signature can provide incremental prognostic value on top of clinical risk scores for the near-term risk of stroke, as was demonstrated in a proof-of-concept study by Han et al. [44]. The ABC [Atrial fibrillation Better Care] pathway has been utilized as a simple yet comprehensive multidisciplinary approach aimed at providing holistic care for AF patients and incorporates AF burden into care [45]. In a post hoc analysis of the AFFIRM [Atrial Fibrillation Follow-up Investigation of Rhythm Management) trial, use of the ABC pathway was associated with a reduction in adverse outcomes in “clinically complex” patients [46]. Utilizing the ABC pathway is projected to prevent a substantial number of strokes and other cardiovascular events [47].

## 4. AF Burden beyond Stroke Risk

**Figure 1:**
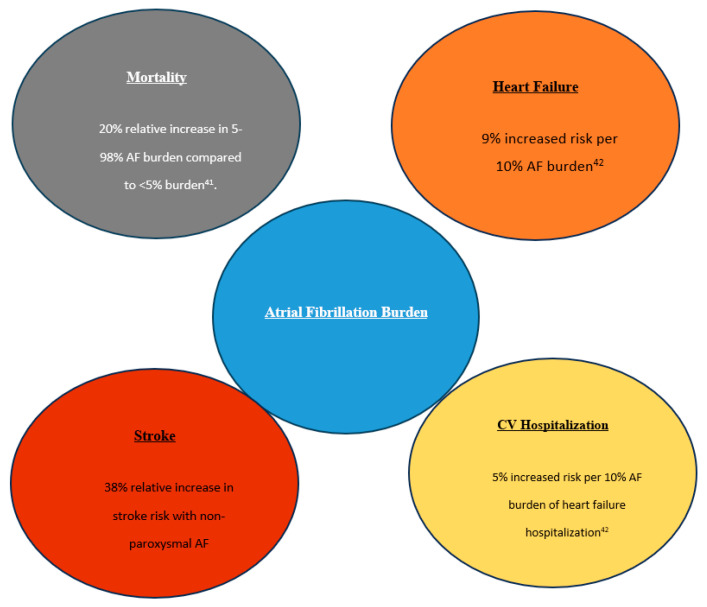
Impact of atrial fibrillation burden on cardiovascular outcomes. Adapted from Chew et al. [41], Steinberg et al. [42], Ganesan et al. [35].

The implication of AF burden extends beyond stroke risk and can affect outcomes such as heart failure, quality of life and health care utilization, cardiovascular hospitalization, cognitive impairment, and mortality (Figure 1). Chew and colleagues demonstrated a clinically relevant dose–response relationship between increasing AF burden and rates of adverse outcomes in patients with paroxysmal AF who had a cardiac implantable electronic device for continuous AF monitoring [48]. The study evaluated 39,710 patients and found that all-cause mortality at the 1-year mark increased with baseline AF burden, 8.54% with AF burden 0%, 8.9% with AF burden 0% to 5%, and 10.9% with AF burden 5% to 98% (*p* < 0.001), as well as an association between increased AF burden and all-cause mortality or cardiovascular hospitalization and ischemic stroke after adjusting for baseline characteristics [48]. Similar results were found at the three-year mark and when accounting for the use of oral anticoagulants.

Using data from the same nationwide database with 39,710 patients with remote monitoring, Steinberg et al. found that in patients without heart failure, AF burden was significantly associated with the increased risk of new-onset heart failure [9% increased risk per 10% AF burden] and all-cause mortality [5% increased risk per 10% AF burden] [49]. In patients known to have heart failure, AF burden was significantly associated with increased risk of heart failure hospitalization [5% increased risk per 10% AF burden] and all-cause mortality [6% increased risk per 10% AF burden] [49].

Other studies have also found similar links between AF burden and heart failure. In a sub-study of the CASTLE AF trial, which evaluated catheter ablation of AF in patients with heart failure and reduced ejection fraction, Brachmann and colleagues found that at the timepoint of 6 months post ablation, an AF burden below 50% was associated with a 67% reduction in primary composite outcome and 77% reduction in all-cause mortality [50]. Their analysis showed that the risk of the primary endpoint or mortality was directly related to a low (<50%) or high (≥50%) AF burden at 6 months post ablation [50].

In another sub-study of CIRCA-DOSE, Andrade and colleagues aimed to determine the AF duration and burden-associated clinical outcomes that were meaningful [51]. AF recurrence limited to durations ≤1 h was associated with rates of healthcare utilization similar to no AF recurrence; AF recurrences lasting >1 h had a 3-fold increase in relative risk for emergency department consultation, 5-fold increase for hospitalization, and 27-fold increase for repeat ablation Similarly, an AF burden ≤0.1% of overall time in rhythm monitoring had rates of healthcare utilization similar to no AF recurrence; AF burden of >0.1% had a two-and-a-half-fold increase in relative risk for emergency department consultation, almost 7-fold increase in risk for hospitalization, 9-fold increase in cardioversion, and 21-fold increase in repeat ablation. AF-related quality of life was significantly impaired with AF episode durations >24 h or AF burdens >0.1% compared to patients who had no AF recurrence [51]. In a separate analysis, using an AF quality of life score, there was a clinically meaningful improvement in quality of life for every 30% relative reduction in AF burden from baseline [52]. In an analysis of the CAPCOST study, patients were found to have significant improvement in quality of life as measured by the same validated scale post ablation, which correlated with a reduction in AF burden [53]. Also, in a sub-study of the STAR AF randomized trial, significant improvements were found in all three ablation strategies used regardless of procedural outcome, and quality of life scores were only adversely affected in patients with a high burden of AF [54]. AF burden also affects activity level; data from DISCERN AF revealed a significant inverse association between activity levels and AF burden. Daily activity progressively decreased after 500 min of AF and then significantly diminished after 1000 min. After adjustment for clinical variables, the association between activity level and AF burden remained statistically significant (*p* = 0.02) [55].

Finally, the association between AF burden and cognitive impairment was recently assessed. Atrial fibrillation has been associated with vascular dementia [56]. In addition, Proietti and colleagues have shown that patients with AF may be exposed to an increased risk of developing new onset of Alzheimer’s disease [57]. In addition, recent evidence suggests that AF and cognitive impairment may be downstream events of atrial cardiomyopathy, which is in turn caused by metabolic syndrome and obesity, suggesting that cardiovascular and metabolic comorbidities may be mediators of the association between AF and cognitive impairment [58]. Tsai and colleagues also found that patients with dementia compared to patients without dementia had a higher risk of incident AF, suggesting a possible bidirectional relationship [59]. Evidence suggests that anticoagulation may protect against or slow the progression of cognitive impairment by reducing silent brain infarctions or microemboli; rhythm control strategies may also be useful in this regard [60]. In the previously mentioned study by Tsai et al., for patients with dementia who experienced new-onset AF, NOAC use was associated with a better clinical outcome compared with no anticoagulation [59]. This is important, as patient with cognitive impairment may be less able to report symptoms of AF or be less considered for NOAC use by treating physicians due to a perceived lower AF burden. Interestingly, the longitudinal burden of AF based on age at diagnosis is significantly associated with the risk of developing dementia [61].

## 5. Lessons Learned from Subclinical AF

**Figure 2:**
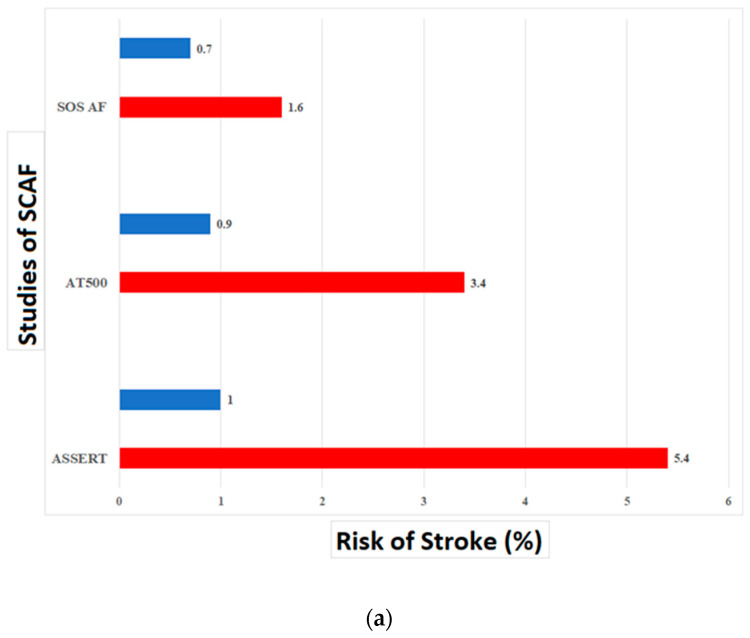

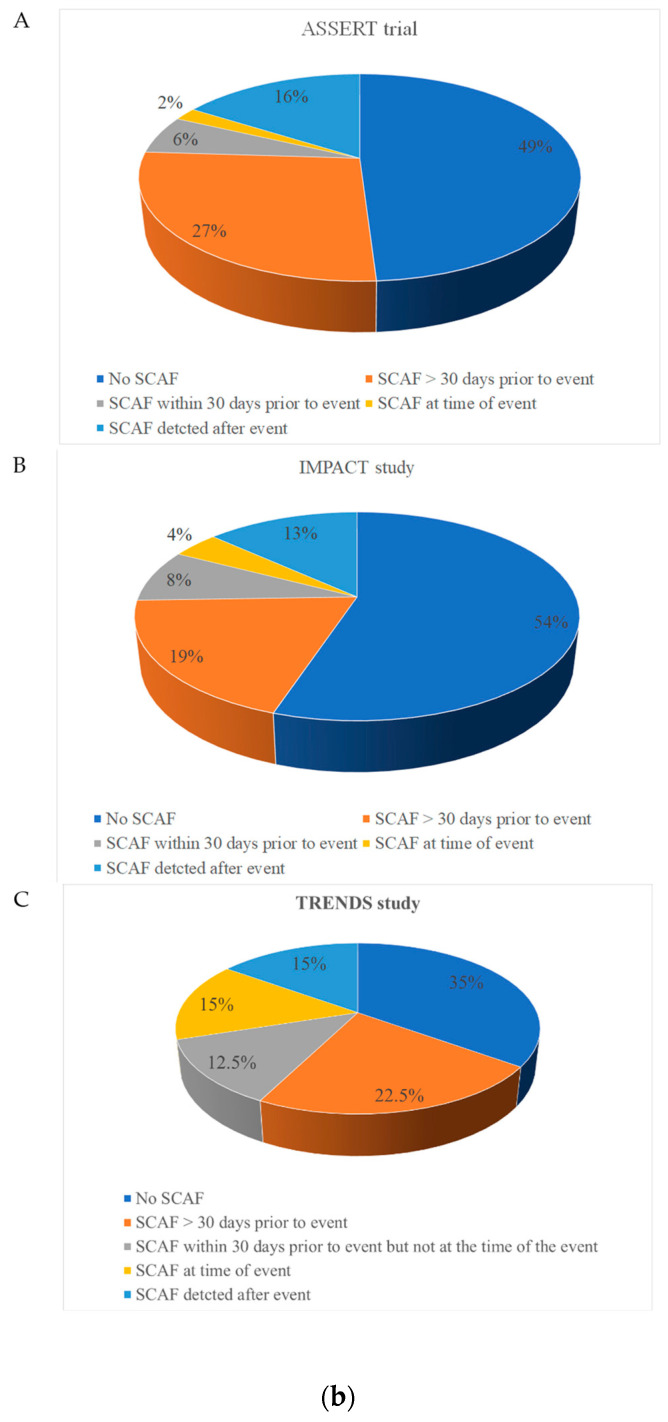
SCAF burden and risk of stroke. (**a**) The risk of stroke associated with a SCAF burden ≥ 23 h in different studies compared to no SCAF. Risk of stroke in patients with SCAF ≥ 23 h (red) compared to no SCAF (blue). (**b**) SCAF and stroke: A temporal relationship based on data from (A) ASSERT, (B) IMPACT, and (C) TRENDS. All stroke or systemic embolism events are correlated with SCAF. SCAF = subclinical atrial fibrillation. Reproduced with permission from AlTurki, A.; Marafi, M.; Russo, V.; Proietti, R.; Essebag, V. Subclinical Atrial Fibrillation and Risk of Stroke: Past, Present and Future. *Medicina* **2019**, *55*, 611 [26].

SCAF is a term used to describe AF detected by cardiac devices in an asymptomatic patient [26,62]. Therefore, in SCAF, even a very low level of AF burden can be detected easily [25]. Given the definition of SCAF as asymptomatic, the most important implication is the risk of stroke and the threshold for anticoagulation. In the ASSERT trial, 2455 patients not known to have clinical AF who had received a cardiac implantable electronic device were monitored for three months for the development of AF, followed by a mean follow-up of two and a half years. At the three-month monitoring period, SCAF had occurred in 10.1% of patients. SCAF was associated with a 5-fold increased risk of clinical AF (*p* < 0.001) and a 2.5-fold increased risk of stroke or systemic embolism (*p* = 0.007), which remained significant after adjustment for stroke risk [62]. The risk of stroke was not elevated to the degree seen with clinical AF, which is four to five times the general population, but was significant at two to two-and-a-half times the risk of the general population [26]. Similar findings were observed in a systematic review in which SCAF strongly predicted clinical AF and was associated with elevated absolute stroke risk that was lower than clinical AF [63]. Proietti and colleagues performed a systematic review to assess the association between SCAF burden and stroke risk (Figure 2), but the authors could not confirm a direct correlation between increasing SCAF burden across the various cut-offs and stroke risks and a small number of studies [64].

Finding the ideal cut-off has been the focus of multiple studies. Importantly, for SCAF studies that predominantly compare AF burden utilizing the longest AF episode, the question of whether this is the optimal stratification method is open to discussion. As stated previously, using the total time in AF likely provides a more comprehensive risk profile.

The group with the data comprises patients with episodes exceeding 24 h. In this population, the risk of stroke is clearly elevated and approximates that seen in clinical AF. Van Gelder and colleagues evaluated data from the ASSERT trial; episodes of AF exceeding 24 h were associated with a significantly increased risk of stroke or systemic embolism (*p* = 0.003) with a similar mean clinical risk profile between groups [65]. In that analysis, including patients with SCAF between 6 min and 24 h, the risk of stroke was not significantly different from patients without SCAF. These findings were corroborated in a systematic review that also found a three-fold increased risk of stroke in SCAF of 24 h or more compared to no SCAF [66].

Data in other SCAF groups in were less clear regarding whether treatment with anticoagulation was warranted. A cut-off of five and a half hours of SCAF was associated with greater stroke risk than no SCAF but was lower than patients with more than 24 h [67].

Episodes of SCAF greater than five to six minutes were also associated with an increased stroke risk, as seen in several studies [62,68]. The limitation to these analyses is as seen in the ASSERT trial; the risk seen in the SCAF greater than six minutes is driven by those with episodes greater than 24 h [62]. It is this equipoise that led to the conduction of two large trials in this group of patients with less than 24 h but greater than five minutes of SCAF [69,70].

Transitioning to a higher SCAF burden is also associated with increased stroke risk. Boriani and colleagues pooled patient level data from three prospective studies with 6580 patients not known to have AF and a mean follow-up period of 2.4 ± 1.7 years. Of those who developed SCAF, 34% had a burden of ≥5 min. Of these patients, 49.8% transitioned to a higher SCAF-burden threshold during follow-up [71]. Approximately 24% of patients transitioned from a lower threshold to a daily SCAF burden of ≥23 h during follow-up. In another ASSERT sub-study, the association between progression to SCAF > 24 h, which occurred in 15.7% of patients, and the development of clinical AF and heart failure hospitalizations was evaluated [72]. The rate of heart failure hospitalization among patients with SCAF progression was 8.9% per year compared with 2.5% per year for those without progression. After multivariable adjustment, SCAF progression was independently associated with HF hospitalization (*p* = 0.004) similar to what is seen with clinical AF [72].

Deciding to initiate anticoagulation in patients with SCAF is challenging due to the issues presented. Given that oral anticoagulation significantly decreases the risk of stroke in clinical AF, the risk reduction would intuitively translate to SCAF, but it is important to remember that the risk of stroke in SCAF overall is around half that seen in clinical AF [26]. Crucially, the risk of stroke in SCAF greater than 24 h is similar to clinical AF, and in this group, anticoagulation is based on the risk profile that is similar to patients with clinical AF.

Two trials were recently reported in patients with a lower SCAF burden. ARTESIA was a prospective, multicenter, double-blind, randomized controlled trial, enrolling patients with SCAF detected by a cardiac implantable electronic device of at least 6 min but less than 24 h and who had additional risk factors for stroke (55 years or older with at least one other risk factor). Important exclusions were clinical AF or other indications for anticoagulation. In the trial, 4012 participants were randomized to receive 81 mg of apixaban or aspirin daily, and some received placebo pills accordingly [69]. The primary outcome was a composite of stroke, TIA, and systemic embolism, and after a mean follow-up of 3.5 ± 1.8 years, stroke or systemic embolism occurred at a rate of 0.78% per patient-year in the apixaban group and 1.24% per patient-year in the aspirin group with a 37% risk reduction (*p* = 0.007). However, the rate of major bleeding was 1.71% per patient-year in the apixaban group and 0.94% per patient-year in the aspirin group, an 80% relative risk increase [70]. In NOAH-AFNET 6, an investigator-driven, prospective, parallel-group, randomized, event-driven, double-blind, multicenter trial, patients 65 years of age or older, who had SCAF lasting for at least 6 min and at least one additional risk factor for stroke, were randomly assigned to receive edoxaban or placebo [70]. The primary efficacy outcome was a composite of cardiovascular death, stroke, or systemic embolism, evaluated in a time-to-event analysis. The analysis population consisted of 2536 patients, but the trial was terminated early, at a median follow-up of 21 months, due to safety concerns and after an assessment for futility. A primary efficacy outcome event occurred at a rate of 3.2% per patient-year in the edoxaban group and 4.0% per patient-year, which was not statistically significant in the placebo group. A safety outcome event occurred at a rate of 5.9% per patient-year in the edoxaban group and 4.5% per patient-year in the placebo group (*p* = 0.03). The main difference in the trials is that ARTESIA used aspirin as the comparator whilst NOAH-AFNET 6 used placebo or aspirin (50%); trial characteristics are summarized in Table 1. Importantly, the primary outcome of both trials was different: stroke or systemic embolism in ARTESIA versus a composite of cardiovascular death, stroke, or systemic embolism, which were evaluated in time-to-event analysis. Patient characteristics in the trials were quite similar. From all the above, the risk of stroke in SCAF greater than 24 h is similar to clinical AF; anticoagulation is necessary, similar to patients with clinical AF. For a duration of less than 24 h, the data are still controversial regarding the question of whether there is an independent risk.

## 6. Future Directions

There is now growing evidence to support the notion of an increased risk of stroke and other cardiovascular outcomes with greater AF burden. However, more research is clearly required to better understand the connection between them and clearly identify the various instances in which intervention, in particular oral anticoagulant use, leads to a net clinical benefit. A key point will be coming to a consensus on the definition of AF burden and whether to continue with using the longest AF episode, as is the case in the SCAF literature, or moving toward percentage of time in AF or the frequency of episodes. Much of this will depend on how long-term monitoring for AF will proceed in the future. The variation in AF burden over time presents another difficulty in quantifying AF burden. Progression to longer episodes occurs not infrequently and is associated with increased risk of cardiovascular outcomes [73]. In the ARTESIA and NOAH-AFNET 6 trials, around 10% of patients progressed to clinical AF [69,70]. Importantly, the interplay between reduction in AF burden and a potential reduction in stroke risk remains a key lingering question. AF ablation clearly reduces AF burden and prevents progression to more persistent forms of AF [14]. Observational data have suggested a decrease in stroke risk post ablation [41], but this has not been corroborated in data from large trials so far. In the CABANA trial, AF ablation reduced AF recurrence by 50% but did not lead to a decrease in the rate of disabling stroke. This was limited, however, by a low stroke rate [0.7% versus 0.1%] and a high rate of crossover to ablation, which precludes from drawing inference regarding a reduction in burden and stroke rate [74]. Further data are needed to explore this avenue, and, as mentioned, the results of the OCEAN trial are eagerly awaited.

## 7. Conclusions

Recent studies in the literature suggest a dose–response relationship between AF burden and stroke risk in both clinical AF and SCAF, which differs from current guidance to disregard burden and utilize clinical risk scores alone. Within clinical classification and at the same risk levels in various scores, the risk of stroke increases, with AF burden opening the possibility to incorporating burden into risk profiles, which has shown promise. Further research is needed to better optimize burden assessment, stroke risk stratification, and anticoagulation in the burden spectrum.

## Figures and Tables

**Table 1:** Clinical trials assessing anticoagulation in patients with subclinical atrial fibrillation.

	ARTESIA [70]	NOAH-AFNET [71]
**N**	4012	2536
**Comparator**	Apixaban versus aspirin	Edoxaban versus placebo or aspirin (50%)
**SCAF definition**	Atrial rate ≥ 175 beats per minute and ≥6 min but <24 h	Atrial rate ≥ 170 beats per minute and ≥6 min
**Population**	≥55 years SCAF detected by implanted device No AF	≥65 years SCAF detected by implanted device No AF
**Inclusion criteria**	CHA2DS2-VASc ≥ 3 or ≥75 years or prior stroke	≥1 risk factors for stroke: Heart failure Hypertension Diabetes mellitus Prior stroke or transient Ischemic attack Vascular disease ≥75 years
**Primary outcome**	Composite of stroke and systemic embolism	Composite of cardiovascular death, stroke, or systemic embolism, evaluated in a time-to-event analysis
**Safety outcome**	Major bleeding	Composite of death from any cause or major bleeding
**Follow-up**	Mean: 3.5 ± 1.8 years	Median: 1.8 years
